# Targeting Systems to the Brain Obtained by Merging Prodrugs, Nanoparticles, and Nasal Administration

**DOI:** 10.3390/pharmaceutics13081144

**Published:** 2021-07-27

**Authors:** Giada Botti, Alessandro Dalpiaz, Barbara Pavan

**Affiliations:** 1 Department of Chemical, Pharmaceutical and Agricultural Sciences, University of Ferrara, Via Fossato di Mortara 19, I-44121 Ferrara, Italy; bttgdi@unife.it; 2 Department of Neuroscience and Rehabilitation—Section of Physiology, University of Ferrara, Via L. Borsari 46, I-44121 Ferrara, Italy; pvnbbr@unife.it

**Keywords:** lipidization, prodrug, influx transporters, active efflux transporters, receptor-mediated transport, micro and nanoparticles, self-assembled nanoparticles, brain targeting, nasal administration

## Abstract

About 40 years ago the lipidization of hydrophilic drugs was proposed to induce their brain targeting by transforming them into lipophilic prodrugs. Unfortunately, lipidization often transforms a hydrophilic neuroactive agent into an active efflux transporter (AET) substrate, with consequent rejection from the brain after permeation across the blood brain barrier (BBB). Currently, the prodrug approach has greatly evolved in comparison to lipidization. This review describes the evolution of the prodrug approach for brain targeting considering the design of prodrugs as active influx substrates or molecules able to inhibit or elude AETs. Moreover, the prodrug approach appears strategic in optimization of the encapsulation of neuroactive drugs in nanoparticulate systems that can be designed to induce their receptor-mediated transport (RMT) across the BBB by appropriate decorations on their surface. Nasal administration is described as a valuable alternative to obtain the brain targeting of drugs, evidencing that the prodrug approach can allow the optimization of micro or nanoparticulate nasal formulations of neuroactive agents in order to obtain this goal. Furthermore, nasal administration is also proposed for prodrugs characterized by peripheral instability but potentially able to induce their targeting inside cells of the brain.

## 1. Introduction

### 1.1. The Physiological Barriers between Bloodstream and Central Nervous System

The drugs currently used against brain diseases constitute about 5% of the drug market and are limited essentially to treating chronic pain, schizophrenia, epilepsy, and depression. On the other hand, major neurological diseases such as cerebrovascular, inflammatory, neurodegenerative diseases or cancers are rarely treated with conventional drugs [1,2,3]. This is mainly due to the frequent inability of drugs to permeate across the structural and functional barriers that protect the central nervous system (CNS), namely the blood brain barrier (BBB) and the blood-cerebrospinal fluid barrier (BCSFB) [1,4]. The BBB segregates the brain interstitial fluid (ISF) from the bloodstream and is constituted by a monolayer of specialized endothelial cells of brain microvessels lined by the basal lamina (basement membrane) and strictly connected by tight junctions (TJs) that hamper the presence of fenestrae. The presence of astrocytes and pericytes complete the BBB, whose role is ISF protection from toxic compounds and exposures to variations in blood composition [1,5,6]. By contrast, the endothelial cells of the capillaries within the choroid plexus are characterized by intercellular gaps and fenestrations. In this case, the ependymal cells of the choroid plexus are strictly connected by TJs, forming the BCSFB, whose role is the secretion and regulation of the cerebrospinal fluid (CSF) [1].

Another important physiological barrier is the blood-retinal barrier (BRB), constituted by retinal capillary endothelial (inner BRB) and retinal pigment epithelial (RPE) cells (outer BRB). TJs of the outer BRB allow it to strictly regulate the flux of molecules into and out of the retina [1,7] similarly to the BBB or BCSFB of the CNS. 

### 1.2. Prodrug Approach for the Brain Uptake of Neuroactive Agents

#### 1.2.1. Lipidization of Neuroactive Agents

Hydrophilic and high molecular weight molecules cannot cross the BBB or BCSFB by paracellular pathways, whereas lipophilic solutes can permeate passively across these types of barriers [1,8]. For this reason, about 40 years ago, the lipidization of hydrophilic drugs was considered a promising approach in order to induce their brain targeting. In other words, hydrophilic drugs were chemically transformed into lipophilic prodrugs by masking polar functional groups with nonpolar and lipophilic substituents. In particular, polar functional groups able to form hydrogen bonds were considered detrimental for the BBB permeation of drugs [9]. The prodrugs obtained by lipidization were designed as inactive agents in vivo able to easily access target tissues (in this case the brain), where their conversion to parent active agents was induced by enzymatic or chemical processes [1,10,11]. This approach was successfully applied to morphine, transformed into its lipophilic prodrug heroin, by double acetylation. This prodrug increased up to 100-fold the BBB permeability in comparison to morphine [12]. Once in the brain, heroin was enzymatically converted to morphine, allowing its interaction with the opioid receptor. Moreover, morphine in its turn did not appear able to diffuse back to the bloodstream across the BBB owing to its hydrophilic properties, according to a “lock-in principle” [12]. Unfortunately, the morphine/heroin example was only one of the few lipidization cases allowing the brain targeting of a hydrophilic drug. Indeed, it was recognized that a lot of lipid-soluble drugs or prodrugs did not permeate into the brain as readily as expected by their lipophilicity. This phenomenon was attributed to the uptake of lipidic molecules in other organs of the body beyond the brain, with consequent reduction of their concentration in the bloodstream and lowering of their passive diffusion power across the physiological barriers [13,14]. 

#### 1.2.2. The Active Efflux Transporters Can Hinder the Brain Uptake of Lipidized Prodrugs

Moreover, at that time, the existence of active efflux transporters (AET) had just begun to be considered. In particular, their expression on the BBB and BCSFB was recognized as very important for brain protection from lipophilic damaging molecules [15]. Taking into account this aspect, lipidization was considered to transform a hydrophilic drug into an AET substrate with consequent rejection from the brain [1,15]. It was indeed known that mice knockout (KO) for the mdr1 gene encoding the efflux transporters P-glycoprotein (P-gp) allowed an enhanced brain distribution of several drugs compared to normal mice [16,17,18], causing their neurotoxicity in KO mice [19,20]. 

Currently, AET systems are known to be members of two transporter gene superfamilies [13,14,21]: the ATP-binding cassette (ABC) gene family (energy-dependent primary active transporters able to efflux their substrates against a concentration gradient) and the solute carrier (SLC) gene family (energy-independent passive transporters). 

The transporters involved in the poor drug intake of the brain are the P-gp, the Multidrug Resistance Proteins (MRP1, MRP4, MRP5) and the Breast Cancer-Resistance Protein (BCRP), as far as the ABC family is concerned, whereas in the SLC group includes the Organic Anion Transporter (OAT3) and the Organic Anion Transporting Polypeptides (OATP1A2). All these transporters, apart from OAT3, are also involved in the multidrug resistance (MDR) of brain tumors [22]. Coordinated activity between ABC and SLC transporters in the BBB or BCSFB cells induces the removal of xenobiotics from the brain [22].

#### 1.2.3. The Design of Prodrugs as Substrates of Influx Transporters of Essential Nutrients

On the other hand, phenomena opposite to drug efflux from the brain have been observed. Indeed, some hydrophilic molecules, such as glucose or vitamin C, were recognized to target the brain very easily, despite their poor lipophilicity. Dopamine (the dopaminergic neurotransmitter used against the Parkinson’s disease) did not appear able to cross the BBB because of its hydrophilic properties, but its l-Dopa prodrug, more hydrophilic than dopamine, was observed to easily enter the brain. These phenomena were attributed to a Carrier Mediated Transport (CMT), enabling solutes such as carbohydrates, amino acids, monocarboxylic acids, hormones, fatty acids, nucleotides, inorganic ions, amines, choline, nutrients, and vitamins to cross the BBB and BCSFB via substrate-specific influx transporters. l-Dopa was recognized as a substrate of amino acid transporters [1,15]. Therefore, the design of prodrugs able to be recognized and transported by the influx transporters was proposed as a valuable strategy alternative to lipidization, to allow drug uptake into the brain [14]. CMTs can be concentrative (ATP-driven or Na^+^-dependent), i.e., aimed at transporting substrates against their gradient of concentration, or equilibrative, which includes simple and/or facilitated diffusion of substrates following their gradient of concentrations and without the requirement of energy [23]. The concentrative CMT of vitamin C (SVCT2), and the equilibrative CMT of l-type amino acid (LAT1) and glucose (GLUT 1), are the most studied in terms of transport of prodrugs [1,8].

#### 1.2.4. The Receptor Mediated Transport and the Brain Uptake of Nanoparticulate Systems

Insulin can be abundantly found in the brain, where its mRNA is absent [24]. The presence in the brain of insulin, as well as other large compounds (peptides or proteins), was explained by the expression on BBB or BCSFB of receptors able to promote endocytotic and transcytotic processes for the transport of large compounds. This phenomenon, called Receptor Mediated Transport (RMT), is induced not only by insulin receptors but also by transferrin or low-density lipoprotein (LDL) receptors [8]. Biodegradable nanoparticles loaded with neuroactive drugs and decorated on their surface with substrates of these types of receptors, were designed to induce their passage across the BBB [25]. A similar transcytotic approach was adopted with nanoparticles decorated on their surface with substrates of the influx transporters LAT1 or GLUT 1 to obtain brain targeting (see Section 4.2) [5,26].

### 1.3. The Focus of This Review

This review shows that the targeting of neuroactive drugs in the brain and its cells can be designed not only with prodrugs able to be recognized and transported by influx transporters, but also with prodrugs able to inhibit or elude the AET systems. Moreover, the prodrug approach is described as very useful to optimize the encapsulation of neuroactive drugs in nanoparticulate systems that can be designed to induce their RMT across the BBB by appropriate decorations on their surface. Essential nutrients able to activate influx transporters are included among these surface features. This review shows that several prodrug approaches can be seriously limited in their ability to induce brain targeting by metabolic peripheral processes and binding with plasma proteins. Taking into account these aspects, nasal administration is proposed as a valuable alternative to obtain brain targeting of drugs. In this regard we describe how the prodrug approach can allow optimization of the nasal administration of neuroactive agents in order to obtain their brain targeting. On the other hand, nasal administration is also proposed as a solution for prodrugs characterized by peripheral problems but potentially able to induce their targeting into the brain cells. 

## 2. Drugs Unable to Permeate in the Brain from the Bloodstream Can Be Transformed into Prodrugs Able to Be Transported by Influx Transporters

Influx transporters show generally high stereospecificity for their substrates, therefore the neuroactive drugs can rarely be recognized and transported across the BBB or BCSFB by these proteins. As a consequence, during the past twenty years, the prodrug approach was proposed to overcome this drawback according to two main strategies: (i) the drug modification into a “pseudonutrients” structure, able to be transported by CMT; (ii) the conjugation of neuroactive drugs with nutrients known to be transported by CMT. In both cases, the drugs were designed to be released after enzymatic cleavage from their prodrugs targeted into the brain or its cells [1,5]. The results of these studies highlight that concentrative transporters, such as SVCT2, are highly selective about the transport of their substrates, offering only limited opportunities to design prodrugs able to be transported. On the other hand, equilibrative transporters, such as GLUT1 or LAT1, appear more versatile in recognizing and transporting appropriately designed prodrugs [1,5,26].

### 2.1. Prodrugs and the Concentrative Transporter of Vitamin C (SVCT2)

**Figure 1:**
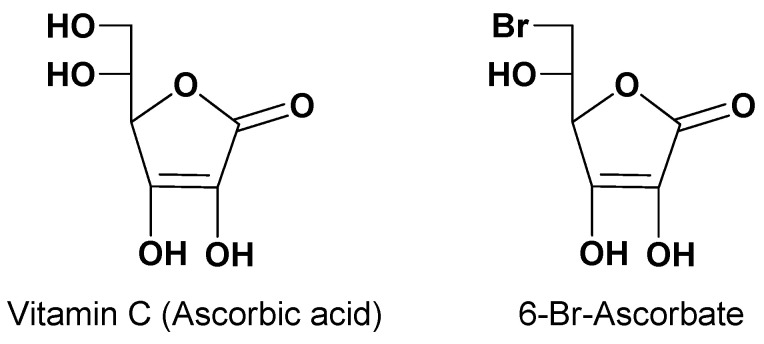
Chemical structures of Vitamin C and 6-Br-Ascorbate.

Vitamin C (ascorbic acid, Figure 1) is an essential nutrient for the spinal cord, brain and eyes of mammals, even if not synthesized in humans [27]. Two concentrative Na^+^ dependent influx transporters of vitamin C (SVCT1 and SVCT2) guarantee vitamin C accumulation inside the body [28,29]. In particular, SVCT1 is located in apical membranes of intestinal cells, whereas SVCT2 is in ependymal cells of the choroid plexus, in the membrane of glial cells and neurons, and in the retinal pigment epithelium [30,31,32]. The activity of these transporters allows the intake of exogenous vitamin C, which in normal conditions can reach concentrations up to 50 μM in the plasma, 500 μM in the CSF, 1 mM in glia and 10 mM in neurons [32]. Taking these aspects into account, the design of prodrugs capable of transport by SVCT2 has shown to be greatly promising for the targeting of neuroactive drugs into the brain. Moreover, the evidence that the derivative 6-Br-ascorbate (Figure 1) was transported by SVCT2 with higher affinity than vitamin C amplified the expectations of this approach. In particular, prodrugs potentially able to be transported into the brain from the bloodstream were designed as ester-conjugates of neuroactive drugs at position 6 of vitamin C [9]. 

**Figure 2:**
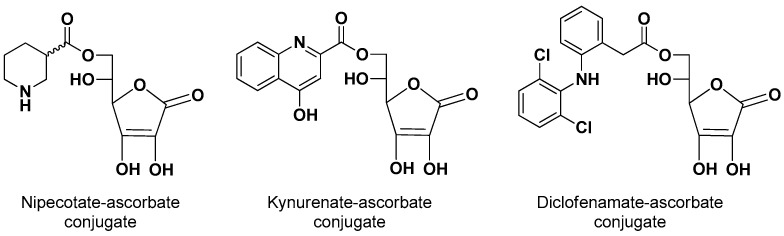
Chemical structures of vitamin C conjugates of nipecotic acid, kynurenic acid and diclofenamic acid.

The 6-ascorbate ester conjugates of nipecotic, kynurenic and diclofenamic acids were synthesized (Figure 2) to evaluate their ability to interact with SVCT2 and to induce therapeutic effects, considering that these drugs were unable to permeate the brain from the bloodstream [9]. 

Human retinal pigment epithelium cells (HRPE) [33] have been widely used as an in vitro model not only of BRB but also of BBB and BCSFB, [34,35,36,37]. In this case, HRPE cells were chosen as a model for the in vitro analysis of the endogenous activity of SVCT2 transporters after demonstrating that they express the SVCT2, but not the SVCT1 transporter on their membrane [38]. Nipecotic and kynurenic acids appeared unable to interact with SVCT2, different from their conjugates that were evidenced as competitive inhibitors of vitamin C, and thus able to interact with its binding site and, therefore, potentially able to be transported by SVCT2. However, only the nipecotic acid conjugate appeared to be transported into the HRPE cells and able to induce neuronal effects following systemic injection in mice. Similar behavior was found with the 5-ester conjugates of the same drugs with 6-Br-ascorbate [35,38]. Diclofenamic acid emerged in HRPE cells as a potent noncompetitive inhibitor of SVCT2-mediated transport of vitamin C, and also able to counteract the in vivo neuronal effects of the conjugates of nipecotic acid [39]. Instead, its prodrugs were evidenced as a competitive inhibitor of SVCT2, unable to permeate the brain [35,36,37,38]. These data suggest that only neuroactive drugs characterized by a relatively small hindrance, such as nipecotic acid, may be suitable for a prodrug-approach for transport by SVCT2 in the brain.

### 2.2. Prodrugs and the Equilibrative Transporters of Sugars (GLUT1, GLUT3 and GLUT5)

The human brain constitutes about the 2% of body weight, but it requires 20% of the glucose consumed in the body, i.e., up to 150 g per day [40,41]. Therefore, it is estimated that the BBB endothelial cells transport an amount of glucose up to ten times their weight per minute to provide the energy required by the brain [42]. The transport of glucose across the BBB is allowed by the equilibrative influx transporter GLUT1, whose preferred substrates are pentose and hexose sugars with a pyranose conformation, including D-glucose [43]. GLUT1 shows a relatively high degree of selectivity for its substrates, even if lower with respect to SVCT2. As a consequence, the chemical transformation of neuroactive drugs into “pseudonutrients” does not appear suitable to obtain prodrug transportable by GLUT1. On the other hand, drug conjugation with glucose has been identified as a valuable strategy to obtain prodrugs able to be transported by GLUT1 [1]. 

Besides the BBB, the cell membrane of the brain parenchyma constitutes a secondary barrier for neuroactive drugs acting on intracellular target sites [44]. Influx transporters located on the membrane of this type of cell may allow the cellular uptake of properly designed prodrugs of neuroactive agents. As equilibrative CMT, GLUT3 is the most important glucose transporter in neurons. Prodrugs properly designed to be transported by both GLUT1 and GLUT3 may constitute a strategy to target neuroactive drugs in the neurons from the bloodstream. GLUT5 is a transporter of furanose located in microglia, unable to transport glucose. GLUT1 is overexpressed in a great amount of cancer cells, including brain tumors, thus it may allow the uptake of prodrugs of antitumor agents [5,45,46]. 

It is described below how the intranasal delivery is an alternative way to achieve uptake into the brain of therapeutics by overlapping the BBB and BCSFB [22] (see Section 5). Therefore, nasal administration of prodrugs able to be transported by GLUT3 or GLUT5 may be considered a strategy to obtain the uptake of neuroactive agents in neurons or glia, respectively.

**Figure 3:**
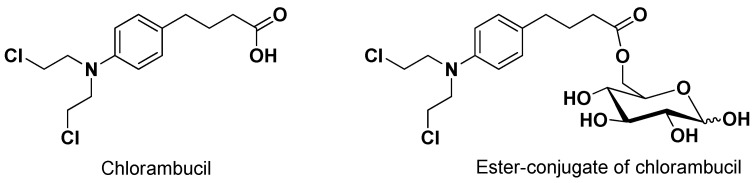
Chemical structures of chlorambucil and its ester conjugate with D-glucose. Despite its ability to interact with GLUT1 with high potency, the prodrug is not transported.

As evidenced for SVCT2, the ability of a prodrug to interact with a transporter does not necessarily imply its transport. Accordingly, the anticancer drug chlorambucil conjugated with D-glucose (6-O-4-[bis(2-chloroethyl)amino]benzenebutanoyl-d-glucopyranose, Figure 3) appeared able to inhibit the uptake of ^14^C-glucose in erythrocytes 160-fold greater than glucose, even if it was not transported by GLUT1 [47].

On the other hand, 7-chlorokynurenic acid, an antidepressant drug unable to permeate the brain, appeared capable of inducing protective effects in mice against seizures after ester conjugation at position 6 of glucose, suggesting GLUT1 involvement for prodrug transport into the brain [48,49].

Dopamine was conjugated at C3 position of glucose via a succinyl linker. This prodrug showed antiparkinsonian effects after systemic administration, suggesting the GLUT1 involvement for its transport into the brain [50]. The ability of GLUT1 to transport this dopamine-glucose was then demonstrated by using HRPE cell line as a model for in vitro transport studies [36].

Ketoprofen and indomethacin conjugation at position 6 of glucose produced prodrugs able to inhibit glucose transport in the brain by GLUT1. This transporter was hypothesized to be involved in the brain uptake of these conjugates [51].

Ester conjugation of ibuprofen was obtained at positions C2, C3, C4 and C6 of D-glucose; such a strategy increased the drug concentrations in plasma of rats after injection of the prodrugs. The conjugate at position C6 induced the highest concentration of the drug in the brain of rats, indicating GLUT1 as a good candidate for the prodrug transport [52].

### 2.3. Prodrugs and the Equilibrative Transporter of L-Type Amino Acids (LAT1)

LAT1 has a lower transport capacity in comparison to GLUT1 [53]. Despite this, LAT1 is one of the most studied CMT for drug uptake into the brain [26]. Indeed, it is highly and selectively expressed in both apical and basolateral sides of the BBB [54]. Moreover, it is also expressed in brain parenchymal cells such as neurons, astrocytes and microglia [26], therefore suggesting LAT1 as potentially involved not only in the brain uptake of prodrugs of neuroactive agents, but also in their internalization into the brain cells [55]. Finally, LAT1 appears overexpressed in several human tumors, including glioma [26]. 

**Figure 4:**
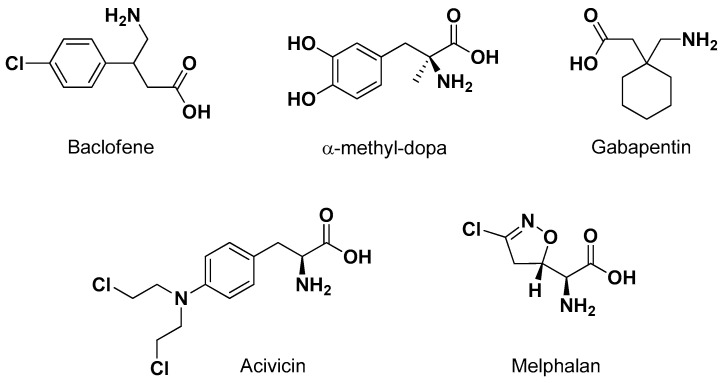
Chemical structures of drugs transported by LAT1.

LAT1 provides the transport of large neutral amino acids, such as L-leucine, L-isoleucine, L-tryptophan and L-phenylalanine [56]. Several studies demonstrated that the necessary structural features allowing a compound to interact with LAT1 are the negatively charged carboxyl and the positively charged amino groups. The affinity for LAT1 appears seriously reduced with any modification in these substituents [57]. Moreover, the presence of a hydrophobic side chain appears important for the transport in the brain by LAT1, in particular when it includes aromaticity [58,59,60]. These structural requirements allowed identification of several drugs as substrates of LAT1. Moreover, LAT1 prodrugs have been designed according to both the “pseudonutrients” and the “conjugates with amino acids” approaches [26]. Accordingly, the neuroactive drugs baclofen (an antispasmodic GABAB agonist), α-methyl-Dopa (a central sympathomimetic used against high blood pressure), gabapentin (an antiepileptic) and the anticancer drugs melphalan or acivicin (Figure 4) were recognized to be transported by LAT1 [26].

**Figure 5:**
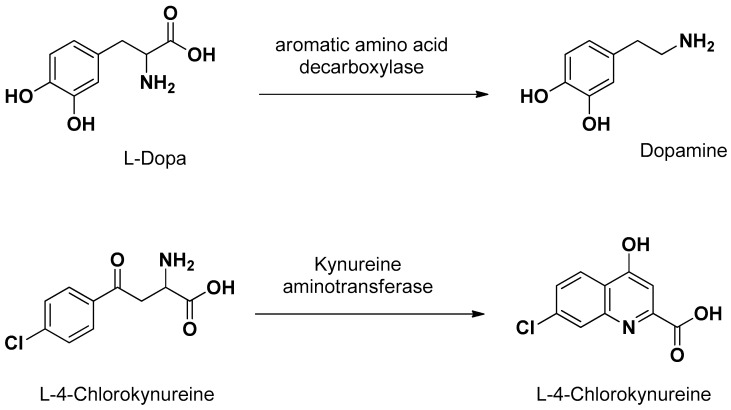
Prodrugs designed as “pseudonutrients” transported by LAT1, and the delivered parent drugs.

Concerning the prodrug approach as “pseudonutrients”, besides l-Dopa, L-4 chlorokynureine (Figure 5) was designed as a prodrug of 7-chlorokynurenic acid transported by LAT1 into the brain, where it is converted to the parent drug by kynureine aminotransferase [61]. In the brain, l-Dopa delivers dopamine by the enzymatic activity of aromatic amino acid decarboxylase (Figure 5) [1].

**Figure 6:**
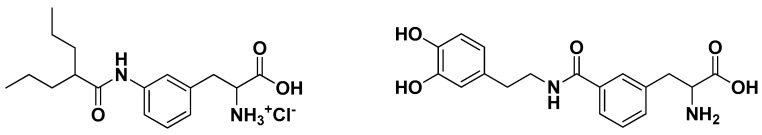
Prodrugs of valproic acid and dopamine obtained by their amide conjugation with phenylalanine. The meta configuration appears optimal for their transport by LAT1.

Valproic acid and dopamine were conjugated to phenylalanine via amide bonds. Brain perfusion techniques demonstrated that the meta configurations of these prodrugs (Figure 6) are optimal in order to promote their LAT1 transport [62,63].

**Figure 7:**
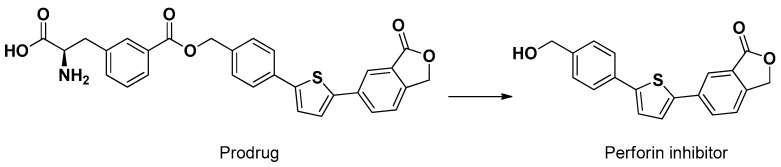
Ester conjugate of an inhibitor of perforin with phenylalanine. This prodrug appears transported by LAT1 into the brain, neurons and astrocytes.

A similar meta configuration was obtained by the ester conjugation of a compound inhibitor of perforin, a pore-forming cytolytic protein, with phenylalanine (Figure 7). This prodrug, different from perforin, was able to penetrate the brain of mice following intraperitoneal injection. Moreover, the conjugate showed an uptake about four times higher than the parent compound in cortical neurons and astrocyte primary cultures [55].

A recent study performed on mouse primary neurons, astrocytes and immortalized microglia (BV2) demonstrated that LAT1 is localized at similar levels in these cells, though astrocytes show the highest affinity for the LAT1 substrate L-leucine, followed by neurons and microglia, in the same way as the perforin conjugate above described. Similar behavior was also evidenced for ketoprofen and ferulic acid conjugates with phenylalanine [3].

Four nonsteroidal anti-inflammatory drugs, flurbiprofen (FLB), salicylic acid (SA), ibuprofen (IBU) and naproxen (NAP), were synthesized as amide meta conjugates of phenylalanine and their uptake was studied in astrocytes and microglial cells. Moreover, the brain distribution of these prodrugs and their parent drugs was analyzed following their intraperitoneal injection in mice. The prodrugs, different from the parent drugs, appeared efficiently transported by LAT1 in the cells. The amide conjugation of the drugs with phenylalanine was chosen to obtain peripheral stability in vivo of the conjugates, avoiding a premature bioconversion that was previously observed for the parent ester conjugates [64]. Following in vivo administration, three prodrugs showed concentration ratios (brain/plasma) higher than their parent drugs (FLB, SA, NAP), whereas the IBU prodrug evidenced the same values of the parent drug. SA and NAP were released into the mouse brain by the prodrugs, in contrast to FLB and IBU, which were not released. The problems related to the FLB and IBU prodrugs were attributed to nonspecific protein binding in plasma, and unexpected peripheral instability, respectively [64]. 

#### Nasal Administration of Prodrugs LAT1 Transported: An Opportunity to Gain the Uptake of Neuroactive Agents in the Cells of Brain Parenchyma?

It is interesting to note that the difficulties described above may be overcome by nasal administration of nonsteroidal anti-inflammatory prodrugs, allowing them to reach the brain by overlapping the BBB and BCSFB [22] (see Section 5). Once in the brain parenchyma, the prodrugs may be transported in the cells by LAT1. Following nasal administration, ester conjugates may also offer useful opportunities. 

## 3. The Prodrug Approach to Elude AETs

We highlighted above that AET systems can limit the permeation of molecules across the BBB and BCSFB, and that the lipidization of hydrophilic molecules can induce their transformation into AET substrates. An attempt to address this problem has been the use of specific inhibitors of efflux transporters. Permeation studies with in vitro cellular models of physiological membranes suggested this approach as a very promising one. On the other hand, the administration of the AET inhibitors was found impracticable at the clinical level. Indeed, the inhibition of AET activity in healthy cells of the body induced severe unwanted effects [22]. In the last ten years, however, new prodrugs have been designed to evade AETs. The prodrug approach was focused in particular on anti-HIV agents that appeared efficacious at the peripheral level but unable to permeate the brain, being substrates of AETs. Instead, HIV was known to easily permeate the brain, where macrophages located in subarachnoid spaces of CSF protect the virus, allowing its replication [65,66] and therefore constituting a sanctuary for HIV from which the periphery is continuously reinfected [67]. The HIV agents normally used in combination antiretroviral therapy (cART) were unable to produce their effects in the brain, being substrates of AETs expressed not only in the BBB [68], but also in the membrane of macrophages [69]. 

### 3.1. The Prodrug Approach with Omo- or Hetero-Dimers of Antiviral Drugs

**Figure 8:**
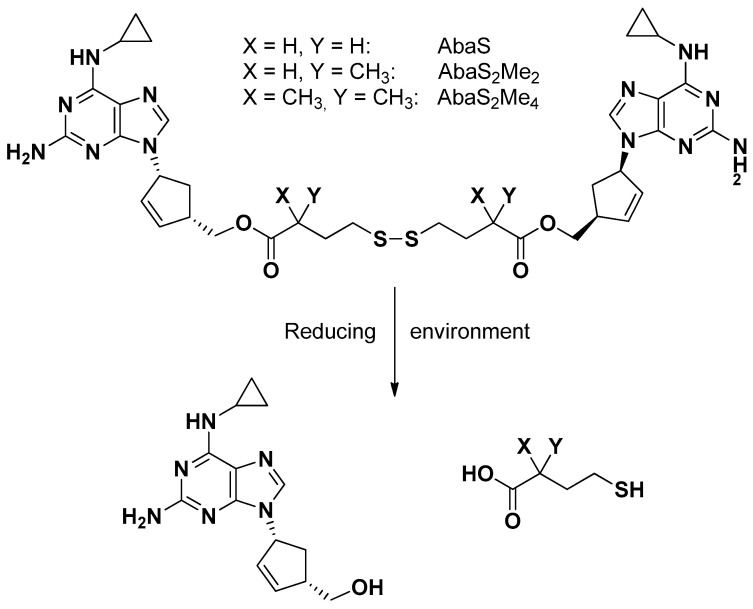
Dimerization of abacavir. The dimers can act as both P-gp inhibitors and prodrugs of abacavir.

The reverse transcriptase inhibitor (RTI) abacavir is known as a substrate of P-gp [70], whose binding site appears constituted by a large cavity, as evidenced by its mouse-solved crystal structure [71]. Dimeric substrates of P-gp, able to interact with this binding site, appeared as potent P-gp inhibitors. Abacavir was, therefore, converted into a potent dimeric P-gp inhibitor by using traceless tethers, as reported in Figure 8, characterized by the presence of a central disulfide group adjacent to two ester linkages to abacavir. These tethers were designed to break down in reducing cell environments, so the dimers were defined both as P-gp inhibitors and prodrugs of abacavir [70].

The inhibitory potency of P-gp drug efflux appeared directly related to the prodrug tether length, whereas its methylation induced the inhibition of ester hydrolysis in plasma but not the release of monomers through the reductive pathway within the cytosol [72,73]. 

A similar approach allowed obtaining dimeric prodrugs of the reverse transcriptase inhibitor abacavir, and one of two protease inhibitors, nelfinavir or darunavir. Again, the dimers appeared as potent P-gp inhibitors in T cells and endothelial cells from the BBB. Moreover, the hetero dimers were able to release the parent drugs in a reducing environment, displaying anti-HIV-1 activity in T cells [74].

The dimeric prodrugs described above appear potentially able to cross the BBB and, once in the brain, to reach therapeutic concentrations in the macrophages that act as HIV reservoirs.

### 3.2. The Prodrug Approach by Conjugation with Ursodeoxycholic Acid

**Figure 9:**
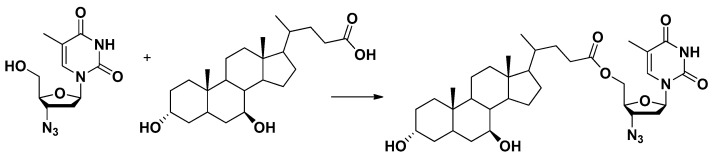
The prodrug of zidovudine obtained by conjugation with ursodeoxycholic acid.

A cell monolayer constituted by HRPE cells was used to demonstrate that zidovudine (AZT), a reverse transcriptase inhibitor, is a substrate of AETs. AZT conjugation with ursodeoxycholic acid (UDCA), a bile acid permeating into the brain, allowed production of a prodrug (UDCA-AZT, Figure 9) able to elude the AETs of HRPE cells without inhibiting them [37]. This behavior should allow the prodrug permeation across physiological barrier expressing AETs without interfering with their activity. Hence, the UDCA-AZT prodrug appears suitable for uptake in macrophages that may constitute the HIV reservoirs. According to this point of view, it has been recently confirmed that UDCA–AZT is able to permeate and remain in murine macrophages with an efficiency twenty times higher than that of AZT delivered in cell environments from the prodrug [69]. As described below (Section 5), the nasal administration of this prodrug allowed concentrations to be reached in the CSF of rats that are potentially suitable for a therapeutic effect on HIV-infected macrophages [69].

## 4. The Use of Nanoparticulate Systems for Drug Delivery into the Brain

Properly designed nanoparticles are able to enter cells by endocytosis or to induce transcytosis and permeate through physiological barriers. These mechanisms differ from those adopted by the free drugs, which diffuse through the cell membranes and becoming susceptible to AET efflux. The nanoparticles can indeed shuttle their loaded drugs away from AET systems, providing their release in cell compartments where the efflux pumps are not expressed, or outside the cells after permeation processes [22]. Therefore, biocompatible and biodegradable nanoparticulate systems seem to offer interesting properties to obtain drug targeting in specific compartments of the body. As described above, the RMT can allow nanoparticles to cross the BBB when decorated on their surface with substrates of receptors expressed by endothelial cells of the brain, such as lactoferrin, transferrin or low-density lipoprotein (LDL) receptors. Apolipoprotein E (ApoE) or its fragments have been proposed as substrates of LDL receptors in order to obtain the BBB permeation of nanoparticulate systems [75,76,77]. 

### 4.1. The Opsonization Problem of the Nanoparticles 

The first types of nanoparticles studied for targeting of their incapsulated drugs were constituted by poly(alkyl cyanoacrylates) (PACA), which are biocompatible and biodegradable polymers [78]. Upon intravenous administration, these nanoparticles appeared to be quickly opsonized by plasma proteins in order to be recognized by the macrophages of the mononuclear phagocytic system (MPS), resulting in their fast clearance from the bloodstream and their accumulation in the Kupffer cells of the liver, phagocytic cells in the spleen and bone marrow [78]. This phenomenon allowed the therapeutic management of chronic intracellular infections of both liver and spleen macrophages by using antibiotic-loaded nanospheres [79,80]. 

The opsonization of the nanoparticles can be very useful for the treatment of diseases related to the MPS, but it can constitute a serious drawback when the nanoparticles are designed for other types of disease, such as brain pathologies. Indeed, their opsonization removes them from the bloodstream before they can reach their designed therapeutic target [22]. This difficulty can be overcome by coating the nanoparticle surfaces with hydrophilic polymers, such as polyethylene glycol (PEG), able to repel the absorption of opsonin proteins. This strategy allows prolonging the circulation times in the bloodstream of the nanoparticles by several orders of magnitude, such that these nanoparticles are named “stealth” nanoparticles [22,81].

### 4.2. Nanoparticles and the Drug Delivery to the Brain

Poly(butyl cyanoacrylate) (PBCA) nanoparticles were the first polymeric nanoparticulate systems studied for drug delivery across the BBB [82]. It was shown that the surface coating of PBCA nanoparticles with polysorbate 80 (Tween 80) allows an increase (up to 20-fold) of their uptake in endothelial cells [82]. The surface coating of nanoparticles with Tween 80 induces the adsorption of plasma ApoE, able to activate the LDL receptors and, therefore, RMT processes across the BBB. A similar mechanism was attributed to poly(lactic-*co*-glycolide) (PLGA) nanoparticles coated with the surfactant poloxamer 188 [22]. PLGA is a biocompatible and biodegradable polymer approved by FDA for human administration [83]. Interestingly, doxorubicin encapsulated in Tween 80-coated PBCA or poloxamer 188-coated PLGA nanoparticles were able to reduce the size of glioblastoma transplanted in rats after intravenous administration [84].

Recently, several nanoparticulate systems have been designed to induce RMT processes across the BBB for the brain targeting of therapeutic agents. In this regard, nanoparticulate systems decorated on their surface with substrates of LAT1 and GLUT1 influx transporters were proposed to promote RMT processes for both the drug uptake in cancer cells and the BBB crossing.

**Figure 10:**
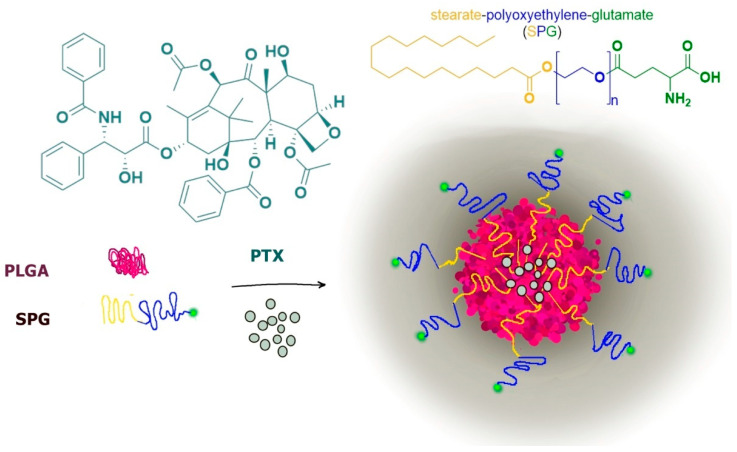
Loaded Paclitaxel (PTX) PLGA nanoparticles modified for LAT1 recognition.

A typical example is constituted by paclitaxel-loaded PLGA nanoparticles functionalized on their surface with glutamine for LAT1 recognition by using polyoxyethylene stearate conjugated with γ-carboxyl of glutamate (SPG). The stearate moiety allowed the SPG insertion in PLGA nanospheres, whereas the external polyoxyethylene-glutamate group allowed the LAT1 recognition, induced by the preserved glutamate free α-amino and α-carboxyl groups (Figure 10).

The LAT1-targeting nanoparticles showed significant increase in cellular uptake and cytotoxicity in tumoral cells (HeLa and MCF-7) with respect to unmodified nanoparticles, evidencing the LAT1 role in these internalization processes. Interestingly, the internalized LAT1 appeared recycled back onto the cell membrane in order to guarantee continuous cellular uptake [85].

**Figure 11:**
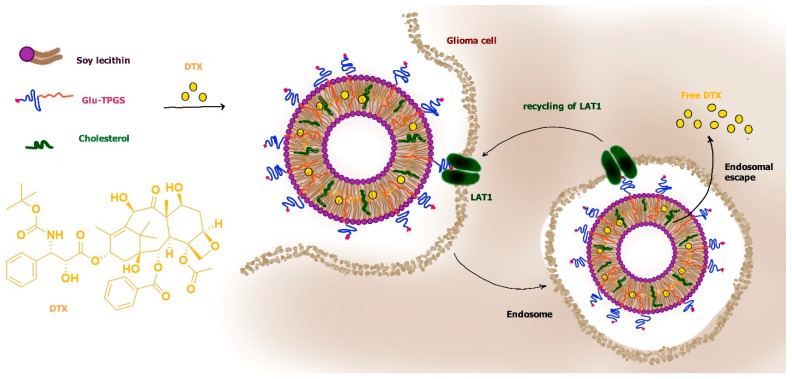
Modified liposomes loaded with Docetaxel (DTX) for BBB crossing and glioma internalization via LAT1.

According to this type of strategy, loaded docetaxel liposomes were modified for LAT1 recognition by inserting d-α-tocopherol polyethylene glycol 1000 succinate to which the γ-carboxyl group of glutamate (glu) was attached (glu-TPGS). The tocopherol moiety allowed the insertion of glu-TPGS in the bilayer of liposomes, whereas glutamate attached to PEG, leaving its free α-amino and α-carboxyl groups, induced LAT1 recognition. The PEG decoration also induced “stealth” properties to liposomes. It was demonstrated that the glu interaction with LAT1 allowed induction of RMT processes of the liposomes across the BBB, allowing their penetration into the brain. Moreover, these liposomes appeared easily internalized in C6 glioma cells via LAT1-dependent RMT processes (Figure 11) [86].

Again, liposomes were designed to inhibit orthotopically implanted mouse glioblastoma growth by loading them with WP-1066, a potent STAT3 inhibitor. The liposomes were modified by inserting in their bilayer both a functionalized amphiphile DOPA (amphi-Dopa) to confer LAT1 recognition, and a 1,2-distearoyl-*sn*-glycero-3-phosphoethanolamine-*N*-amino(polyethylene glycol) (DSPE-(PEG)-amine) to confer “stealth” properties. The modified liposome evidenced LAT1-mediated uptake in glioma (GL261) cells and selective accumulation in brain tissue after intravenous administration in mice, allowing for an increase in their overall survival [87]. 

**Figure 12:**
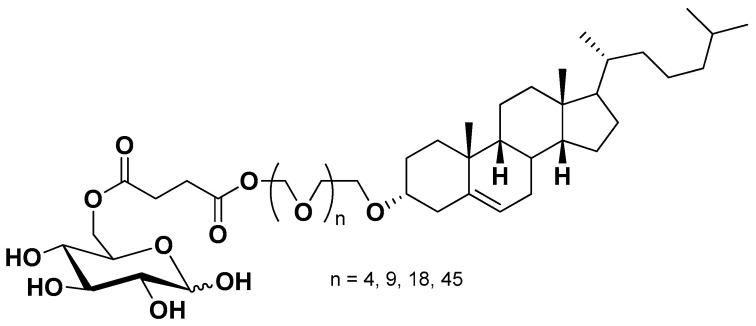
Glucose-cholesterol conjugates used for the formulation of liposomes functionalized for GLUT1 recognition.

Similar strategies were also reported for nanoparticulate systems functionalized on their surface for GLUT1 recognition in order to obtain BBB crossing and glioma drug delivery. As an example, decorated liposomes were prepared by using glucose-cholesterol conjugates via succinic-polyethylene glycols linkers (Figure 12). The cholesterol moiety allowed the insertion of the conjugates in the bilayer of liposomes, whereas glucose attached to polyethylene glycol induced GLUT1 recognition. These decorated liposomes were able to cross a BBB model and exhibited brain delivery capacity in mice [5,88]. 

Recently, 2-deoxy-d-glucose-modified poly(ethylene glycol)-*co*-poly(trimethylene carbonate) nanoparticles (_D_Glu-NP) loaded with paclitaxel were formulated as a drug delivery system to enhance the BBB penetration via GLUT-mediated transcytosis and improve the drug uptake in glioma via GLUT-mediated endocytosis. The modified nanoparticles were able to cross a BBB model and penetrate into RG-2 glioma cells by endocytosis with higher amounts than nonglucosylated nanoparticles. _D_Glu-NPs showed specific and efficient accumulation in intracranial tumor [89].

### 4.3. The Prodrug Approach to Enhance the Encapsulation Efficiency of Neuroactive Drugs in Micro or Nanoparticles

The small size and large surface area of nanoparticles can limit the loading of drugs and cause their relative high burst release. In this case, the encapsulated drugs may be released from the nanoparticles before reaching their therapeutic target in the body. This phenomenon can often occur when the nanoparticles are hydrophobic (for example PLGA or solid lipid nanoparticles) and the encapsulated drugs are hydrophilic or poorly hydrophobic. On the other hand, it is known that hydrophobic prodrugs, when properly designed, can solve these difficulties, allowing appropriate encapsulation efficiencies and adequate release control. 

As an example, the adenosine A_1_ receptor agonist N^6^-cyclopentyladenosine (CPA), identified as a promising anti-ischemic agent but unable to cross the BBB, was efficiently encapsulated in PLGA nanoparticles only as a 5′-ester-octanoyl conjugate CPA prodrug [90,91,92]. Similarly, dopamine appeared unable to be encapsulated in solid lipid microparticles (SLMs), whereas its prodrug 3,4-*O*-divaleroyl-dopamine showed satisfactory encapsulation efficiency and release control [93]. Again, zidovudine was encapsulated in PLGA nanospheres and efficiently controlled for its release only as its UDCA-AZT prodrug (see Section 3.2) [94]. Similarly, the same prodrug allowed zidovudine encapsulation in SLMs, whose nasal administration induced selective SNC targeting [95]. Very recently, the volatile geraniol was efficiently encapsulated in solid lipid nanoparticles (SLNs) only as a prodrug obtained by its ester conjugation with ursodeoxycholic acid (GER-UDCA). In this case, the nasal administration of the nanoparticles induced selective SNC targeting of the prodrug [96] (see Section 5).

A singular prodrug approach was proposed for the formulation of nanoparticles designed for the delivery of a neuropeptide to the brain after oral administration. In particular, a prodrug of leucine5-enkephalin (Tyr-Gly-Gly-Phe-Leu) was obtained by the esterification of the free phenolic hydroxyl group of tyrosine residue with palmitic acid. In aqueous media this prodrug formed nanoparticulate aggregates with quaternary ammonium palmitoyl glycol chitosan. Moreover, the prodrug showed higher stability to plasma peptidases than leucine5-enkephalin, which was further increased upon encapsulation. The nanoparticles loaded with the prodrug were able to promote the brain uptake of leucine5-enkephalin after oral administration [97]. It has been hypothesized that the encapsulated prodrug would be protected from degradation within the gut, and that the mucoadhesive properties of the nanoparticles (due to the chitosan presence, able to interact with the negative charges of mucous layers with its positively charged amino groups [98]) allowed enhancement of the prodrug oral absorption. Moreover, it was further proposed that the absorbed prodrug would then be able to cross the BBB because of its greater plasma stability and lipophilicity [97]. It would be interesting to verify if this prodrug may be able to elude AET systems. 

In order to obtain the encapsulation in nanostructured lipid carriers, apomorphine was transformed into its lipophilic prodrug diacetyl or diisobutyryl apomorphine. The nanoparticulate systems showed a brain distribution after intravenous administration [99].

Methotrexate cannot be encapsulated in SLN, due to its hydrophilicity. For this reason, a prodrug was designed as a lipophilic ester, obtaining didodecylmethotrexate that was easily encapsulated in nanoparticles based on behenic acid. The nanoparticles were functionalized with a chimera peptide characterized by the presence of an aminoacidic sequence for the very low-density lipoprotein (VLDL) receptor able to target the BBB [100]. Tested on glioma cell lines, the loaded nanoparticles were more effective than the free drug to inhibit their proliferation. In vivo biodistribution studies indicated the important role of chimera peptide in inducing brain targeting of the nanoparticles after intravenous administration [100].

Lipophilic prodrugs of kiteplatin were encapsulated into SLNs based on cethylpalmitate and decorated on their surface with PEG by using 1,2-dipalmitoyl-*sn*-glycero-3-phosphoethanolamine-*N*-[methoxy(polyethylene glycol)-2000] (16:0 PEG2000 PE) during formulation processes. The nanoparticles were obtained with their surfaces coated by Tween 80 in order to induce ApoE absorption and thus promote their BBB crossing by LDL receptor-mediated transcytosis. The presence of PEG on the surface of nanoparticles confer on them “stealth” properties. The nanoparticles appeared able to permeate an in vitro model of the BBB, the human brain microvascular endothelial hCMEC/D3 cell line, and promote the prodrug uptake in human glioblastoma cells [101].

A novel phenanthroindolizidine anticancer, CAT3, is known to inhibit the hedgehog signaling pathway, thus inducing strong inhibitory effects on cancer cells. In particular, the inhibitory effect on temozolomide-resistant glioblastoma multiforme in orthotopic glioblastoma mice models was attributed to CAT3 following its oral administration [102]. This effect could be mainly exerted on cancer stem cells that normally lead to recurrent disease and MDR associated with tumors [81]. CAT3 is a class IV drug in the biopharmaceutics classification system, insoluble in water and with scarce lipid solubility, showing very poor bioavailability following oral administration. Therefore, CAT3 was conjugated to oleic acid through ionic bonding to overcome its insolubility in lipids and to encapsulate it in SLNs based on glyceryl behenate. The nanoparticles were able to increase the CAT3 permeability across intestinal cell monolayers and to enhance oral bioavailability in rats. Moreover, the nanoparticles were able to decrease the viability of C6 glioma cells [103].

### 4.4. The Design of Prodrugs Self-Assembling in Water to Form Nanoparticulate Systems

Traditional nanoparticulate systems, such as polymeric nanoparticles, liposomes or inorganic systems, have been widely used to deliver drugs into the brain, such as lipophilic, hydrophilic, or unstable drugs, but many disadvantages derive from their use [6]. First, they require the presence of carriers to be designed, namely polymers and surfactants, which have to be nontoxic and biocompatible. Moreover, the efficiency of nanocarriers depends on their drug loading, defined as the quantity of drug that the system is able to incorporate and deliver, which can be very poor. Finally, they can lack stability when administered in vivo, which can cause the unwanted release of the drug far from the action site, leading to important side effects and toxicity.

About fifteen years ago, a revolutionary strategy was proposed to design very high loading nanoparticles with increased therapeutic activity in comparison to the free drugs [104]. This opportunity was discovered with a prodrug of gemcitabine (an anticancer drug [105]) obtained by its covalent conjugation with squalene, an acyclic triterpene precursor in the cholesterol biosynthesis. The prodrug obtained was highly lipophilic, appearing able to self-assemble in water to form nanoparticles without the need of a carrier material [78]. This strategy, named “squalenoylation”, was applied to a broad range of different therapeutic agents increasing their potential therapeutic efficacy [78]. As an example, the gemcitabine-squalene nanoparticles obtained by nanoprecipitation were able to increase the in vivo half-life of the anticancer drug, exhibiting improved therapeutic efficacy. In particular, the biosimilarity of squalene with cholesterol induced the interaction of nanoparticles with the circulating lipoproteins acting as substrates of the LDL receptors, whose over-expression in cancer cells allowed the selective intracellular accumulation of the nanoparticulate systems in several tumors [106,107]. 

Doxorubicin-squalene nanoparticulate systems were able to both significantly prolong the drug circulation time in the bloodstream and reduce its cardiac accumulation [108]. This property was attributed to an elongated shape of the nanoparticles, preventing them from being captured by macrophages [108]. 

These interesting results triggered further studies of the squalenoylation approach to adenosine, known for its capacity to stimulate neuronal survival, even if characterized by a very short half-life in vivo and by the inability to permeate into the brain from the bloodstream [109,110]. Squalene-adenosine nanoparticles were able to increase the adenosine half-life in vivo and induce high neuroprotective effects on preclinical animal models of cerebral ischemia and spinal cord injury [111]. Unexpectedly, the nanoparticles appeared unable to cross in vitro models of the BBB, so the neuroprotective effects were attributed to an improvement of cerebral microcirculation due to a vasodilatory action mediated by the released adenosine on endothelial cells and pericyte/astrocytes receptors [78].

Very recently, the prodrug obtained by the ester conjugation of zidovudine with ursodeoxycholic acid (UDCA-AZT, see Section 3.2) has demonstrated the ability to self-assemble as nanoparticle cores coated with a bile acid salt (taurocholate or ursodeoxycholate) corona without any other excipients [112]. The taurocholate-coated nanoparticles appeared able to interact with serum proteins, differently from the ursodeoxycholate-coated particles. Accordingly, the taurocholate-coated nanoparticles showed in vitro uptake by murine macrophages about 70 times higher than that obtained with the free prodrug, whereas no significant uptake increase was registered for ursodeoxycholate-coated particles. Zidovudine was also detected in macrophages following the prodrug uptake, the greatest amounts being produced by taurocholate-coated nanoparticles [112]. These results would seem to suggest that it is possible to modulate the uptake of nanoparticles in macrophages by choosing different bile acid salts during the nanoprecipitation procedures. As a consequence, taurocholate-coated nanoparticles may be useful against intracellular infections of the MPS system, whereas ursodeoxycholate-coated particles could have “stealth” properties in the bloodstream. Ursodeoxycholate-coating may be proposed as a choice alternative to PEG anchorage on the surface of nanoparticles (see Section 4.1) in order to confer them “stealth” properties. This new opportunity may overcome the problems related to PEG layers, such as immune reactions following repeated dosages that can induce accelerated blood clearance of nanoparticles [113,114] and the risk of accumulation of PEG chains in the body with related toxicity [114], as also recognized for the polymers (such as PLGA) that constitute the nanoparticles [115].

Recently, the prodrug self-assembling strategy was further developed by using new prodrugs able to self-assemble into nanoparticulate systems in an aqueous environment and designed with specific linkers showing both high stability in the bloodstream and the ability to release their parent drugs at the site of action, limiting the potential off-target effects of the administered drugs. Some of these formulations are described below. 

#### 4.4.1. The Use of the Disulfide Linker to Release the Drug of Self-Assembled Nanoparticles in the Target Site

An anticancer prodrug was designed by connecting through a disulfide-triazole linker the lipophilic drug camptothecin (CPT) and the hydrophilic drug gemcitabine (GEM) [116], based on a CPT-GEM prodrug self-assembled nanomicellar system previously synthesized [117]. 

Due to its amphiphilic nature, the prodrug CPT−ss-triazole−GEM can self-assemble to form spherical nanoparticles without the help of polymeric or lipidic carriers. Moreover, the presence of a protonated group represented by the 1,2,3-triazole allowed a stable dispersion of the nanoparticles in water, leading to an efficient, nontoxic, biocompatible, and safe drug-delivery system. Indeed, the disulfide group can prevent the accidental release of the entire quantity of drug. As described in Section 3.1 this bond can be very stable in the bloodstream, since it is not hydrolyzed by esterases, but is easily cleaved in glutathione (GSH)-reducing environments such as the cellular cytoplasm of tumor cells. Moreover, the drug loading of self-assembled nanoparticles was very high when compared to traditional nanoparticulate systems, the prodrug itself being the unique component of the nanostructures. The system was designed to reach the tumoral tissues by passive targeting through the enhanced permeability and retention (EPR) phenomenon, which is essentially due to large spaces between endothelial cells of tumor vessels and impaired lymphatic drainage caused by compression of intratumor lymphatic vessels [81]. Interestingly, CPT-ss-triazole-GEM nanoparticles showed a higher cytotoxicity against HepG2 cells when compared to not self-assembled CPT and GEM [116].

**Figure 13:**
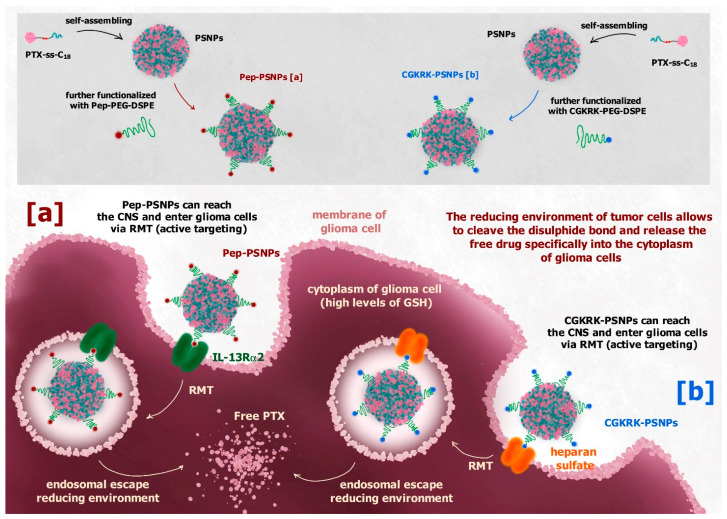
(**a**) PTX-ss-C_18_ prodrug of paclitaxel (PTX) can self-assemble as nanoparticles (PSNPs) that upon functionalization of their surface with Pep-PEG-DSPE (Pep-PSNPs) target glioma cells via interleukin 13 receptor α2-mediated endocytosis, releasing PTX into the glioma cells. (**b**) Upon functionalization of the PSPNPs surface with CGKRK-PEG-DSPE, CGKRK-PSNPs can enter glioma cells via heparan sulphate-mediated endocytosis. In both cases, the reducing environment of tumor cells allows the cleavage of the disulfide linker and the release of the free drug into glioma cells.

Although the system described above is not meant to reach the brain, its design strategy appears very interesting, and was developed to obtain safe drug-self delivery systems able to cross the BBB or the blood-brain tumor barrier (BBTB), thus reaching the brain. As an example, a disulfide linker was used to design a redox-sensitive paclitaxel (PTX) prodrug against glioblastoma multiforme (GBM) able to self-assemble into nanoparticles [118]. Different from the CPT-ss-triazole-GEM prodrug, the hydrophobic PTX was bound to the hydrophobic octadecanol (C_18_) through the disulfide linker. The prodrug PTX-ss-C_18_ appeared able to self-assemble into nanoparticles (PSNPs), which were further functionalized on their surface with PEG and Pep-1 (glioma homing peptide), specific for brain and GBM targeting via interleukin 13 receptor α2-mediated endocytosis (Figure 13a). In vitro studies of functionalized nanoparticles (Pep-PSNPs) on U87MG and BCEC cells showed a higher cytotoxicity against tumoral cells compared to PTX alone, whereas no toxicity was recorded for healthy cells. Moreover, in vivo studies on intracranial glioma-bearing mice models demonstrated the ability of Pep-PSNPs to accumulate into the tumor site, specifically following intravenous administration, and to exhibit a significant improvement in antiglioma efficacy, compared to the drug alone [118]. 

Active targeting can be obtained by decorating the surface of nanoparticles with substrates of transmembrane receptors overexpressed by cancer cells, such as albumin, folic acid, somatostatin or transferrin [81]. In this regard, the same type of self-assembled nanoparticles described above was functionalized on its surface with the CGKRK peptide (CGKRK-PSNPs), a ligand of the heparan sulfate, which overexpresses on the glioma cells membrane, in order to have an active targeting to the glioma site via heparan sulphate-mediated endocytosis (Figure 13b) [119]. The in vitro results were comparable to those obtained for Pep-PSNPs. In this case the CGKRK-PSNPs were combined with borneol (Bor) when administered in vivo. Bor is a traditional Chinese medication used to transiently open the intercellular TJs at the BBB level, improving the permeation of drugs into the brain [119]. 

#### 4.4.2. ROS-Sensitive Linkers of Self-Assembled Prodrugs

A polymer prodrug of paclitaxel (PTX) has been synthesized via conjugation to PEG through a p-(boronic ester)benzyl-based cleavable linker (PEG-B-PTX). This prodrug appeared able to self-assemble in nanoparticulate systems and to release PTX in response to elevated reactive-oxygen species (ROS) levels [120]. The spacer was demonstrated to be very stable and hardly hydrolysable in normal physiological conditions, but oxidizable by the elevated ROS levels present in tumors, releasing the free drug into the tumoral cells specifically, where the ROS levels are elevated. 

Similarly, the anticancer drug doxorubicin (DOX) was conjugated to PEG via a ROS-responsive thioketal moiety to obtain an amphiphilic polymer prodrug able to self-assemble as nanoparticles (PEG-DOX). This nanoparticulate system was designed with “stealth” properties in order to reach the tumor site via the EPR effect and release the free drug in response to the high ROS levels [121]. 

The above-described systems were not thought of as capable to cross the BBB; however, it is interesting to observe that the spacer chosen to create the prodrug self-assembled nanoparticles could be different and sensitive to various environments, thus extending the potentiality of these systems. For example, elevated ROS levels can be found not only in tumoral cells, but also in neurodegenerative diseases, such as Alzheimer’s or Parkinson’s. This aspect introduces the possibility to create safe and efficient drug self-delivery systems free of carriers and characterized by elevated drug loading that can be functionalized to cross the BBB, reach the brain, and release the drugs specifically at their site of action. 

#### 4.4.3. Polymer Prodrug Self-Assembled Nanoparticles

Heterotelechelic polymer prodrugs can constitute alternative substrates allowing functionalized self-assembled nanoparticles including neuroprotective drugs. In this case, the drug of interest is covalently conjugated to a biocompatible polymer used to constitute the nanoparticles. The polymer, in turn, is covalently linked to reactive groups than can be exploited for the conjugation to specific targeting ligands, for example proteins, which allow the system to cross the BBB via RMT and reach the brain. Very recently, a heterotelechelic polymer prodrug of adenosine (Ade) conjugated to a maleimide-functionalized polyisoprene (Ade-PI NPs) was proposed. The reactive group, represented by the maleimide moiety, was exploited for protein conjugation to albumin (BSA), α2-macroglobulin (α2M) and fetuin A, which are known to cross the BBB in healthy physiological conditions via RMT [25]. In comparison with unconjugated nanoparticles, only those conjugated with α2M were able to increase their in vitro permeation across the BBB hCMEC/D3 cell line. Unfortunately, this permeation increase was registered only in the absence of Ade conjugated to the polymer, whereas the drug presence on the surface of nanoparticles induced perturbations on the interaction of α2M with its cell membrane receptors, hindering the RMT phenomenon. This result indicates that the choice of drugs and proteins conjugated to heterotelechelic systems is crucial in order to design efficient targeting systems [25]. 

Polymer prodrug nanoparticles can also be used for sustained release of anticancer drugs. Very recently, PEGylated poly(lactide)-poly(carbonate)-doxorubicin nanoparticles (DOX-NPs) have been synthetized, exploiting a carbamate moiety on the sidechain of the monomer unit to conjugate the drug, which can be successively released by the action of ureases. Their efficient cytotoxic activity on both primary cancer cell lines derived from adult patients following neurosurgical resection, and the commercially available GBM cell line, U87, has been demonstrated [122]. Interestingly, DOX-NPs have further been incorporated in various poly(dl-lactic-*co*-glycolic acid) and poly(ethylene glycol) (PLGA/PEG) matrixes. This strategy allowed the production of thermosensitive in situ gelling PLGA/PEG microparticles. When mixed with saline solutions at room temperature, these microparticles form a paste, enabling the surgeon to line the resection cavity site following removal of the tumor. In this case, the aim is not to obtain a system able to cross the BBB, but, instead, to obtain a depot formulation able to release the DOX-NPs, exploiting their ability to selectively deliver the drug into the tumor cells, avoiding both the entry of the drug into healthy cells, and off-target side effects [122]. 

## 5. Nasal Administration as an Alternative Way to Target the Brain: The Opportunities Offered by the Prodrug Approach

We evidenced above that the permeation of neuroactive drugs into the brain from the bloodstream can be seriously limited by several factors, such as BBB permeation, metabolic peripheral processes, and binding with plasma proteins. Some of these problems can also involve prodrugs designed for brain targeting. The intranasal route can offer an effective alternative to intravenous or oral administrations, in order to obtain drug or prodrug brain targeting [123]. Indeed, this strategy, which is characterized by a higher patient compliance, can often offer a direct nose-to-brain delivery of the molecules allowing bypass of all problems related to the bloodstream. The first concept of the intranasal route for the delivery of therapeutically active compounds directly into the brain was discovered and proposed about 30 years ago by William H. Frey II [124]. This approach was then developed and is currently applied for the brain uptake of low molecular weight neuroactive drugs [125], proteins [126] and cells [127]. Several neuroprotective agents have been studied concerning their brain delivery nasally, including anti-ischemic, anti-inflammatory, anti-Parkinson, antiepileptic and antimigraine drugs [96,128,129,130,131,132,133,134,135], as well as antibiotics [136,137,138] and antivirals [68].

Three regions divide the nasal cavity: the vestibular, respiratory and olfactory. After nasal administration, the drug amounts that are able to escape filtration by nasal hairs in the vestibular region can reach the other two regions, where the drug can be absorbed into the body. The vestibular region is characterized by a limited surface coated by squamous epithelial cells that strongly limit the absorption of drugs. The respiratory region is the largest area of the nasal cavity, and is highly vascularized with blood vessels, which allow the drug molecules to permeate in the bloodstream across the respiratory mucosa. Here, four types of cells constitute the respiratory epithelium: basal cells, goblet cells, nonciliated columnar cells and ciliated columnar cells. These epithelial cells are closely connected, being surrounded by intercellular junctions. Goblet cells contribute to creating the mucus layer that is moved by the coordinated movement of cilia towards the pharynx. Therefore, the mucus layer is able to capture many molecules, effecting their elimination from the nasal cavity. Only the drug molecules able to cross the mucus layer can be absorbed in the bloodstream. In particular, the columnar cells are characterized by the presence of microvilli that increase their surface allowing easy absorption of drugs at the systemic level by a transcellular pathway [125,139]. 

The olfactory region is situated in the upper portion of the nasal cavity, its position allowing the entry of olfactory sensory neurons of the brain. The olfactory epithelium is a modified form of respiratory epithelium constituted by three cell types: basal cells, supporting epithelial (sustentacular) cells and olfactory receptor cells. Bowman’s glands are present in this epithelium providing the secretion of mucus that covers the epithelial surface. The olfactory mucosa is not provided with cilia, even if mucus clearance is allowed by its incessant excretion and gravitational sliding [125,139,140]. Here, the drug molecules able to pass the mucus layer can permeate across the olfactory mucosa and directly reach the CSF or the brain parenchyma. Two main pathways allow the uptake of drugs to the brain from the olfactory region: (i) transcellular permeation across sustentacular cells by passive diffusion or receptor mediated endocytosis; (ii) paracellular permeation through tight junctions between sustentacular cells and olfactory neurons [125]. An extracellular pathway can also allow the drugs to move externally along the length of the neuronal axon of olfactory cells in the brain parenchyma [139].

A small portion of trigeminal neurons is also included in the respiratory and olfactory mucosa, which can contribute to the drug uptake into the brain [123]. To summarize, three main pathways can allow the drug access into the brain from the nose: (i) absorption in the bloodstream (respiratory region) and subsequent BBB crossing (systemic pathway), which may be proposed for potent drugs able to permeate the BBB but characterized by poor oral bioavailability; (ii) direct permeation in CSF across the olfactory mucosa, or transcellular transport via olfactory neurons (olfactory pathway); (iii) transport via trigeminal nerves (trigeminal pathway). The drug delivery from the nose to the CSF or brain parenchyma along the olfactory or trigeminal pathways can occur within a few minutes following an extracellular route without undergoing axonal transport or binding to any receptor.

The olfactory pathway allows for drug distribution in rostral brain structures, including the olfactory bulb, anterior olfactory nucleus, frontal cortex and hippocampus. The trigeminal pathway allows for drug distribution in caudal brain structures, including the upper cervical spinal cord, midbrain, pons and hypothalamus [141].

Alternatively, after endocytosis, some drugs can be axonally transported into the brain. This process, however, requires up to 48 h [68,123].

**Figure 14:**
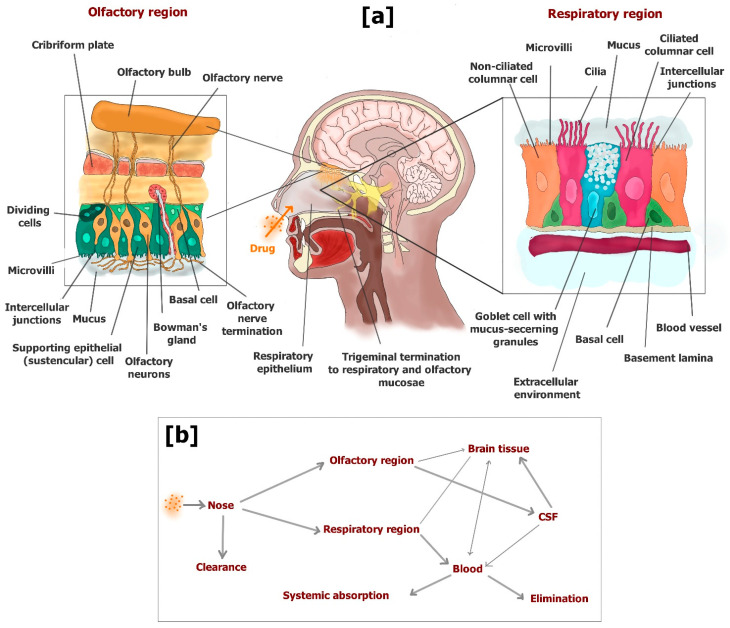
(**a**) Cross section of the human nasal cavity showing the respiratory and olfactory regions and schematic illustrations of the various cell types in their epithelia. A small portion of trigeminal neurons is also included in the respiratory and olfactory mucosa. (**b**) Upon nasal administration, a drug deposited on respiratory and olfactory mucosa can permeate to the bloodstream and liquid cerebrospinal fluid, respectively. Transport via olfactory and trigeminal neurons allows the drug uptake in different regions of brain parenchyma.

Figure 14 shows a scheme of the mechanisms of drug uptake in the brain from the nasal cavity.

In general, the brain uptake of nasally administered drugs is allowed by appropriate formulations able to provide several advantages, such as drug deposition on the olfactory mucosa, an adequate residence time despite mucus clearance and high local concentration of the free drug or prodrug for diffusion processes. According to these requirements, micro or nanoparticulate systems have been designed as solid nasal formulations in order to obtain satisfactory encapsulation efficiency and drug release showing a high burst effect. As an example, solid microparticles based on chitosan salts have been formulated with these properties to obtain an efficient permeation across nasal mucosae of an anti-ischemic drug and an antibiotic [128,138]. Indeed, the chitosan salts dissolved rapidly with water contact, allowing a fast release of the encapsulated drugs. Moreover, chitosan is known for its ability to form a positively charged hydrogel interacting with negatively charged mucosal surfaces. These interactions produce mucoadhesion properties that prolong the residence time on the nasal mucosa of the formulation [142]. Again, the chitosan can transiently open the TJs of mucosa, allowing a transiently increased permeability of the drugs or prodrugs across the nasal mucosa [143]. Taking into account these aspects, nasal formulations based on chitosan nanoparticles have been designed for the treatment and prevention of Alzheimer’s disease [144,145]. Recently, SLMs were designed for a fast release of resveratrol, proposed as neuroprotective agent. In order to obtain a nasal formulation characterized by an efficient nose-to-brain delivery of resveratrol, the SLMs were coated with chitosan [131].

Alternatively, cyclodextrins are considered efficacious promoters of drug permeation across the mucosa, being able to induce perturbation of their bilayer integrity by forming inclusion complexes with phosphatidylcholine, cholesterol and sphingomyelin [146]. Nasal formulations based on cyclodextrins have been proposed in order to obtain an efficient nose-to-brain delivery of neuroactive agents [129,134,147].

### The Prodrug Approach in the Nose-to-Brain Pathway

The prodrug UDCA-AZT (see Section 3.2) is hydrolyzed in brain homogenates [37], indicating its ability to release zidovudine into brain compartments [37]. Moreover, this prodrug has shown evidence of its ability to elude the AET systems, different from its parent drug [37]. As a consequence, UDCA-AZT appeared able to permeate macrophages with an efficiency twenty times higher than zidovudine [69]. A nasal formulation of UDCA-AZT based on chitosan microspheres allowed a direct delivery of the prodrug in the CSF of rats, where its presence was prolonged in time. The ability of the prodrug to elude the AET systems probably contributed to prevent its extrusion into the bloodstream and to induce its permeation in the macrophages (potential HIV sanctuaries) located in the subarachnoid spaces of the CSF [69]. 

The hydrophilic properties of zidovudine hamper its encapsulation in solid lipid micro or nanoparticles. By contrast, the highly lipophilic prodrug UDCA-AZT can be efficiently encapsulated in SLMs. Indeed, SLMs based on stearic acid appeared able to efficiently encapsulate this prodrug and to provide a significant increase of its dissolution rate in water. The nasal administration of this formulation allowed a direct nose-to-brain delivery of UDCA-AZT, which was detected in the CSF of rats. Moreover, in the presence of chitosan these SLMs allowed an increase in prodrug amounts detected in CSF [95].

The high lipophilic properties of UDCA-AZT allowed its self-assembling as nanoparticle cores coated by taurocholate or ursodeoxycholate (see Section 4.4). The particles coated with ursodeoxycholate showed a much higher uptake by murine macrophages than those of ursodeoxycholate-coated particles or free UDCA-AZT. For this reason, the ursodeoxycholate coated nanoparticles were nasally administered and, in the presence of chitosan, produced a satisfactory nose-to-brain delivery of the prodrug [112]. 

Nanoparticles were hypothesized as systems that may be able to permeate the nasal mucosae [148]. Interestingly, it has been demonstrated that nanoparticulate systems can cross, in the presence of chitosan, the TJs of epithelial monolayers by a paracellular pathway [149]. Very recently, this phenomenon has also been evidenced on cell models of intranasal epithelium [150] and in vivo [151]. The possibility that the UDCA-AZT nanocores, or similar nano self-assembled systems, may penetrate the CSF following nasal administration and may open new perspectives in the fight for HIV eradication from the body.

**Figure 15:**
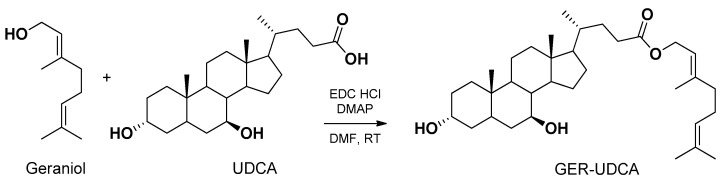
The prodrug obtained by ester conjugation of geraniol with ursodeoxycholic acid.

Geraniol is a natural compound proposed for the treatment of Parkinson’s disease [152]. Even if geraniol is characterized by a relative high lipophilicity, its volatility hampers its encapsulation in polymeric or solid lipid nanoparticles [96]. The nasal administration of geraniol as a water suspension caused serious damage to the nasal mucosae [96]. It is known that ursodeoxycholic acid (UDCA) is effective in rescuing mitochondrial function in Parkinson’s disease patients [153]. Taking into account these aspects, a new prodrug (GER-UDCA) was obtained by the ester conjugation of geraniol with UDCA (Figure 15).

GER-UDCA was able to hydrolyze in brain homogenates and was easily entrapped both into lipid (SLNs) and polymeric (NPs) nanoparticles. The SLNs were able to increase the dissolution rate of GER-UDCA, so they were selected for nasal administration to rats. GER-UDCA-SLNs did not damage the structural integrity of the nasal mucosa and induced a direct nose-to-brain delivery of the prodrug, suggesting their potential suitability for the treatment of Parkinson’s disease patients [96].

In Section 2.3 we evidenced that prodrugs designed to be transported by LAT1 may evidence problems related to their stability and nonspecific protein binding in the bloodstream. It may be useful to verify if the nasal administration of these prodrugs could allow their uptake in the brain where they can potentially permeate brain cells.

Very recently, it has been demonstrated that quinidine, chosen as substrate model of P-gp, is poorly adsorbed in the brain by the nasal pathway, but its brain absorption can be significantly increased by systemic P-gp inhibition, confirming that the uptake of drugs in the brain from the nasal cavity can be influenced by P-gp activity [154]. The nasal administration of the formulations based on the prodrug approach, which was proposed in order to elude the AET systems, can open interesting applications for drug targeting in the brain.

In Section 4.4.1. CGKRK-PSNPs were described as a strategy to specifically deliver the anticancer drug PTX into the brain to obtain an antiglioma effect. These nanoparticles were administered in vivo in combination with Bor, which can transiently slacken the TJs of the BBB and improve permeation into the brain of CGKRK-PSNPs [119]. However, this method is not considered very safe since the BBB is fundamental to maintain brain integrity and avoid damage caused by potentially toxic substances. Alternatively, it may be interesting to verify if this type of functionalized nanoparticle can directly reach the brain upon nasal administration. This strategy should allow bypass of the BBB and, therefore, avoid the use of Bor at the systemic level. Taking into account that in the presence of chitosan nanoparticulate systems have been shown to cross the nasal mucosa, this strategy appears very feasible [150,151].

## 6. An Overview of the Prodrug Approach

Table 1 summarizes the prodrug approaches described in this review for brain targeting. Anti-Alzheimer, anti-Parkinson, central sympathomimetic, antiepileptic, neuroprotective, antivirals, anti-ischemic and antitumor drugs are among the neuroactive agents taken into account, evidencing the need for new strategies to deal with brain diseases. The prodrug approaches include both the opportunity to involve active efflux systems for brain targeting and to elude the AET systems. The formulative approaches appear mainly focused on nanoparticulate systems able to induce RMT processes across the BBB. Among these, self-assembled nanoparticles appear promising for clinical trials, being obtained in the absence of polymeric or lipidic excipients. The new prodrugs and their formulations have been tested in vitro by using cellular models able to simulate the BBB. In vivo tests were performed mainly on rodents in order to analyze the distribution of the drugs and their potential therapeutic effects. Few clinical trials have been currently addressed, mainly on dopamine prodrugs for Parkinson’s disease and other types of drugs (such as anticancer or antiepileptic drugs) as LAT1 pseudonutrients. The main challenges for clinical translation appear focused on the stability of the prodrug at the peripheral level and their ability to deliver neuroactive agents in the brain or in its cells. In this regard it is not guaranteed that the pharmacokinetic and distribution of the prodrugs in human bodies will be the same as detected in rodents. The nasal formulations of prodrugs may constitute an innovative resource for brain targeting of neuroactive agents in humans considering the opportunity to overcome all the difficulties related at the peripheral level.

## 7. Conclusions

About 40 years ago lipidization appeared as one of the first prodrug approaches for brain targeting of neuroactive agents. Even if promising, this strategy was weakened by the activity of active efflux transporters (AETs). Therefore, new approaches were proposed considering the prodrugs as substrates of influx transporters of essential nutrients. In particular, prodrugs of neuroactive agents were successfully proposed for the brain targeting as substrates of LAT1 or GLUT transporters of amino acids or sugars, respectively. Moreover, prodrugs able to inhibit or elude the AETs were also being studied, allowing the targeting in the brain and neutrophils of antiviral drugs. An alternative approach to elude the AETs was proposed considering drug encapsulation in biocompatible nanoparticles. Indeed, properly designed nanoparticles can induce the receptor-mediated transport (RMT) phenomenon, allowing cell uptake or permeation via endocytotic or transcytotic pathways. Nanoparticles functionalized on their surface with substrates of LAT1 and GLUT1 influx transporters were demonstrated to induce RMT phenomena, allowing BBB crossing and permeation in tumor cells. In this case, the prodrug approach appeared important to increase the encapsulation of drugs in the nanoparticles and control their release. About 15 years ago, a revolutionary prodrug approach (squalenoylation) allowed the production of self-assembling nanoparticles, characterized by the absence of carrier excipients and very high amounts of the encapsulated drug. These systems were successfully proposed for the targeting of tumor cells, then the self-assembling strategy was further developed with the use of disulfide linkers, allowing prodrugs that are potentially stable in the bloodstream and easily cleaved in cellular environments. Self-assembled nanoparticles obtained with this type of prodrugs appear able to cross the BBB or target tumor cells upon appropriate functionalization of their surface with RMT substrates.

About 30 years ago, nasal administration of drugs emerged as a valuable strategy to obtain brain targeting of neuroactive agents, bypassing the BBB. Indeed, it was demonstrated that appropriate formulations allow a direct nose-to-brain pathway following their nasal administration. Currently, the prodrug approach appears useful both to increase the loading in nasal micronized or nanonized formulations of neuroactive agents and prevent their extrusion from the brain into the bloodstream. Moreover, once in the brain, properly designed prodrugs appear potentially able to target its specific cells. Finally, the opportunity to design nanoparticles able to permeate the nasal mucosa has recently emerged. This aspect opens intriguing opportunities to obtain brain targeting of neuroactive agents with prodrugs able to self-assemble as nanoparticulate systems.

## Figures and Tables

**Table 1:** Disease specific prodrug approaches for brain targeting of neuroactive agents.

Specific Disease	Formulative Approach	Tested Formulation	Target	Experimental Model	Refs.
Alzheimer’s Disease	LAT1/Lat1-substrate prodrugs	Ketoprofen (nonsteroidal anti-inflammatory), ferulic acid (natural anti-oxidant), immunosuppressive perforin inhibitor prodrugs	Drug uptake into brain parenchymal cells via LAT1	In vitro cultures of mouse primary cortical neurons, astrocytes and immortalized microglia (BV2); in vivo C57BL/6 J mice model of AD	[3,55]
	GLUT1–substrate produgs	Prodrugs obtained as ester conjugates of ibuprofen at C-2, C-3, C-4, and C-6 positions of D-glucose	GLUT1 for BBB crossing	In vitro stability in rat plasma and brain tissue extracts and in vivo rat systemic injection	[52]
	SLMs coated with chitosan	Resveratrol loaded in SLMs	Nose-to-brain delivery	In vitro permeation studies across human normal colon mucosa NCM460 cells; in vivo intravenous and nasal administration to rats	[131]
Parkinson’s disease	Pseudonutrient	l-dopa as prodrug of dopamine	LAT1 for BBB crossing	Used in clinical practice	[1,26]
	GLUT1-substate prodrug	Prodrugs obtained as dopamine-glucose conjugates via succinyl linker	GLUT1 for BBB crossing	In vitro HRPE cell line; in vivo model of rat with morphine induced locomotion and reserpine-induced hypolocomotion	[36,50]
	SLNs	SLNs loaded with a geraniol prodrug obtained by ester conjugation with ursodeoxycholic acid	Nose-to-brain delivery	In vitro incubation in rat brain and rat liver homogenates; in vivo intravenous and nasal administration to rats	[96]
	Lipidic nanocarriers	Apomorphine prodrugs loaded in nanostructured lipid carriers	BBB crossing	In vitro stability tests in human blood samples; in vivo systemic administration to mice	[99]
Central sympathomimetic	Pseudonutrient	α-Methyl-dopa as LAT1 substrate	LAT1 for BBB crossing	Used in clinical practice	[26]
Epilepsy	Pseudonutrient	Gabapentin as LAT1 substrate	LAT1 for BBB crossing	Used in clinical practice	[26]
	SVCT2-substrate prodrugs	Prodrugs of nipecotic acid obtained as ester conjugates of ascorbic acid and 6-Br-ascorbate	SVCT2 for BCSFB crossing	In vitro HRPE cell lines; intravenous injection to pentylentetrazol treated mice	[35,38,39]
	GLUT1-substate prodrugs	Prodrugs of 7-chlorokynurenic acid obtained as glucose ester conjugates	GLUT1 for BBB crossing	In vitro co-cultures of mouse cortical neurons and astrocytes and in vivo model of NMDA-induced seizures in mice	[48,49]
Brain disorders	Liposomes decorated with glucose	Liposomes with glucose-cholesterol conjugates via succinic-polyethylene glycols linkers;	GLUT1 for BBB crossing via RMT	In vitro BCEC and astrocytes cell lines; in vivo systemic administration to mice	[88]
	Nanoaggregates	NPs obtained as aggregates of leucine^5^-enkephalin prodrug with quaternary ammonium palmitoyl glycol chitosan;	Improvement of oral bioavaila-bility, plasma stability and brain uptake	In vitro studies of brain and liver homogenates of rats; in vivo intravenous and oral administration to mice	[97]
	GLUT1-substrate prodrugs	Prodrugs obtained as ester conjugates of ketoprofen and indomethacin with D-glucose	GLUT1 for BBB crossing	In situ rat brain perfusion technique	[51]
HIV-induced neurological disorders	Prodrug able to elude the AETs	Prodrug of zidovudine obtained as ester conjugate with ursodeoxycholic acid	Ability to elude the active efflux o systems	In vitro HRPE cell model	[37]
	Nasal MPs	Zidovudine prodrug loaded in chitosan MPs	Nose-to-brain-delivery	In vitro studies on J774A.1 murine macrophages; in vivo intravenous and nasal administration to rats	[69]
	Nasal MPs	Zidovudine prodrug loaded in solid lipid MPS	Nose-to-brain-delivery	In vivo intravenous and nasal administration to rats	[95]
	P-gp inhibitors	Prodrugs of abacavir obtained as dimeric P-gp inhibitors	BBB crossing via P-gP inhibition	In vitro 2D7-MDR cells (CD4+ human T-lymphocytic cell line expressing P-gp); Insect Sf9 cells expressing P-gp; hCMEC/D3 cell line; brain capillaries freshly isolated from rats	[70,72,73,74]
	Self-assembled NPs	NPs obtained with a prodrug of zidovudine and coated with taurocholate	Nose-to-brain delivery and uptake in macrophages	In vitro studies on J774A.1 murine macrophages; in vivo nasal administration to rats	[112]
Stroke	NPs obtained with heterotelechelic polymers and decorated with α2M	NPs obtained in the presence of adenosine	BBB crossing via RMT	In vitro permeation across hCMEC/D3 cells	[25]
Glioma	Pseudonutrient	Melphalan as LAT1 substrate	LAT1 for BBB crossing	Used in clinical practice	[26]
	SLNs	SLNs loaded with a prodrug of PF403 (CAT3) conjugated to oleic acid	Enhancement of oral bioavailability of CAT3 and brain targeting	In vitro U251/TMZ and T98G cells and C6 glioma cells; in vivo U251/TMZ orthotopic and T98G subcutaneous xenograft rat models; pharmacokinetics in rats	[102,103]
	Self-assembled NPs	NPs obtained by self-assembling a prodrug of paclitaxel and decorated with Pep-1	BBB crossing via RMT	In vitro glioma U87MG cells and rats brain capillary endothelial BCEC cells, in vivo glioma-bearing mice models	[118]
	Self-assembled NPs	NPs obtained by self-assembling of a prodrug of Paclitaxel and decorated with CGKRK peptide	Active targeting to glioma cells	In vivo intravenous administration on glioma-bearing mice models combined with borneol	[119]
	PBCA and PLGA NPs surfactant coated	NPs loaded with doxorubicin	BBB crossing via RMT	In vivo intravenous administration to rats	[84]
	Liposomes decorated with glutamate	Docetaxel loaded in the liposomes	BBB crossing and glioma internalization via RMT induced by LAT1	In vitro C6 glioma cells; in vivo intravenous administration to mice	[86]
	Liposomes decorated with l-Dopa with stealth properties	WP-1066 (STAT3 inhibitor) loaded in liposomes	BBB crossing and glioma internalization via RMT induced by LAT1	In vitro GL261 glioma cells; in vivo intravenous administration to mice	[87]
	Polymeric NPs decorated with 2-deoxy-d-Glucose	Paclitaxel loaded in the NPs	BBB crossing and glioma internalization via RMT induced by GLUT	In vitro RG-2 glioma cells; in vivo intravenous administration to glioma bearing mice models	[89]
	SLNs decorated with a chimera peptide of VLDL	Prodrug of methotrexate loaded in SLNs	BBB crossing via RMT	In vivo intravenous administration to glioma bearing rats	[100]
	SLNs surfactant coated with stealth properties	Prodrugs of kiteplatin	BBB crossing via RMT induced by LDL repcetor	In vitro permeation across hCMEC/D3 and uptake studies in U87-MG cells	[101]
	Thermosensitive in situ gelling polymeric MPs	Polymer prodrug of doxorubicin NPs loaded in thermosensitive MPs	In situ application	In vitro primary cancer cell lines derived from adult patients following neurosurgical resection, and glioma U87 cell	[122]

AD = Alzheimer ‘s disease; BBB = blood brain barrier; BCSFB = blood cerebrospinal fluid barrier; MP = microparticle; NP = nanoparticle; SLM = solid lipid microparticle, SLN = solid lipid nanoparticle; RMT = receptor mediated transport; α2M = α2-macroglobilin; PF403 = phenanthroindolizidine anticancer; VLDL = very low-density lipoprotein.
